# Oxidative Stress Biomarkers and Systemic Inflammatory Indices in Metabolic Dysfunction-Associated Steatotic Liver Disease with Type 2 Diabetes Mellitus: A Comparative and Longitudinal Analysis

**DOI:** 10.3390/ijms27125432

**Published:** 2026-06-16

**Authors:** Vlad Pădureanu, Lidia Boldeanu, Anca Bobîrcă, Diana Clenciu, Rodica Pădureanu, Adina Mitrea, Veronica Gheorman, Ștefan Pătrașcu, Beatrice Elena Vladu, Albert Georgescu, Ionela Mihaela Vladu, Virginia Maria Radulescu

**Affiliations:** 1 Department of Internal Medicine, University of Medicine and Pharmacy of Craiova, 200349 Craiova, Romania; vlad.padureanu@umfcv.ro (V.P.); veronica.gheorman@umfcv.ro (V.G.); 2 Department of Microbiology, University of Medicine and Pharmacy of Craiova, 200349 Craiova, Romania; lidia.boldeanu@umfcv.ro; 3 Department of Internal Medicine and Rheumatology, ‘Carol Davila’ University of Medicine and Pharmacy, 020021 Bucharest, Romania; anca.bobirca@umfcd.ro; 4 Department of Diabetes, Nutrition and Metabolic Diseases, Faculty of Medicine, University of Medicine and Pharmacy of Craiova, 200349 Craiova, Romania; diana.clenciu@umfcv.ro (D.C.); ionela.vladu@umfcv.ro (I.M.V.); 5 Department of Surgery, University of Medicine and Pharmacy of Craiova, 200349 Craiova, Romania; stefan.patrascu@umfcv.ro; 6 Faculty of Medicine, University of Medicine and Pharmacy of Craiova, 200349 Craiova, Romania; beatricevladu75@gmail.com (B.E.V.); albertgeorgescu99@gmail.com (A.G.); 7 Department of Medical Informatics and Biostatistics, University of Medicine and Pharmacy of Craiova, 200349 Craiova, Romania; virginia.radulescu@umfcv.ro

**Keywords:** MASLD, oxidative stress, MDA, 8-iso-PGF2α, NLR, MLR, PLR, FIB-4, type 2 diabetes, liver fibrosis, systemic inflammation

## Abstract

Metabolically dysfunction-associated steatotic liver disease (MASLD) complicated by type 2 diabetes mellitus (T2DM) represents a clinically aggressive phenotype associated with accelerated hepatic fibrosis progression. The interplay among oxidative stress, systemic inflammation, and the risk of hepatic fibrosis in this context remains incompletely characterised. We conducted a single-centre observational study enrolling 110 adult MASLD patients, stratified into two groups: Group 1 (G1, n = 20), patients with concurrent T2DM, followed longitudinally at three successive time points, and Group 2 (G2, n = 90), non-diabetic controls. Serum oxidative stress biomarkers were assessed using malondialdehyde (MDA) and 8-isoprostaglandin F2α (8-iso-PGF2α). Systemic inflammatory status was quantified through the neutrophil-to-lymphocyte ratio (NLR), monocyte-to-lymphocyte ratio (MLR), and platelet-to-lymphocyte ratio (PLR). Hepatic fibrosis risk was estimated using the FIB-4 index. Diabetic MASLD patients exhibited significantly elevated levels of 8-iso-PGF2α (*p* = 0.014) and NLR (*p* = 0.016) compared with controls, indicating greater oxidative membrane damage and systemic neutrophilic inflammation. A robust inverse correlation between PLR and FIB-4 was observed across all analytical strata (combined cohort: Spearman r = −0.680, *p* < 0.001). MLR emerged as the only independent predictor of MDA in G1 (β = 841.78, *p* = 0.013). Longitudinal analysis demonstrated biomarker stability over time, except for a significant increase in ALT from T1 to T2 (*p*_adj = 0.014). These findings support the clinical utility of routinely available haematological inflammatory ratios and lipid peroxidation biomarkers for phenotypic characterisation of MASLD in the diabetic context, highlighting the need for larger prospective studies with histological validation.

## 1. Introduction

Metabolic-dysfunction-associated steatotic liver disease (MASLD)—redefined through a landmark multisociety Delphi consensus in 2023 as the replacement for the former term nonalcoholic fatty liver disease (NAFLD)—is currently the most prevalent chronic liver disease worldwide [1]. Its diagnosis requires the presence of hepatic steatosis in conjunction with at least one of five cardiometabolic risk factors, including overweight/obesity, impaired fasting glucose or type 2 diabetes mellitus (T2DM), arterial hypertension, hypertriglyceridemia, or reduced HDL cholesterol [1,2]. Affecting approximately 38% of the global adult population [3,4], MASLD encompasses a histological spectrum ranging from simple steatosis to metabolic dysfunction-associated steatohepatitis (MASH), progressive fibrosis, cirrhosis, and hepatocellular carcinoma (HCC), constituting a major contributor to liver-related morbidity and mortality worldwide.

The coexistence of T2DM and MASLD is epidemiologically and pathophysiologically significant. The global prevalence of MASLD among individuals with T2DM is estimated at 55–70% [5], and diabetes is consistently identified as one of the strongest independent predictors of disease progression to advanced fibrosis and MASH [6]. In patients with T2DM, fibrosis progression is accelerated compared to non-diabetic individuals, and HbA1c independently correlates with hepatic fibrosis stage [6]. This clinically important comorbidity substantially amplifies the risk of cirrhosis, portal hypertension, and liver transplantation eligibility, translating into a growing socioeconomic burden on healthcare systems [7].

At the molecular level, the pathogenesis of MASLD—and its acceleration by diabetes—is inextricably linked to oxidative stress. Chronic hyperglycemia promotes overactivation of the mitochondrial electron transport chain, leading to excessive reactive oxygen species (ROS) generation [8]. These ROS overwhelm endogenous antioxidant defence mechanisms, initiating lipid peroxidation cascades that produce secondary aldehyde products, most notably malondialdehyde (MDA) [9,10]. MDA—the principal end product of polyunsaturated fatty acid peroxidation—is one of the most widely used clinical biomarkers of oxidative lipid damage [10], and its levels have been reported to correlate with both liver disease severity and glycemic control in T2DM patients [11]. 8-isoprostaglandin F2α (8-iso-PGF2α), a prostaglandin-like compound formed exclusively via non-enzymatic arachidonic acid peroxidation, represents a more specific and chemically stable marker of systemic lipid peroxidation [12], with demonstrated elevations in both MASLD [9] and T2DM contexts [8,11].

Mechanistically, chronic hyperglycemia and insulin resistance promote mitochondrial electron transport chain overload, excessive superoxide generation, activation of the polyol pathway, advanced glycation end-product formation, protein kinase C activation, and NADPH oxidase stimulation. These convergent pathways amplify reactive oxygen species production and initiate lipid peroxidation of polyunsaturated fatty acids within cellular and mitochondrial membranes. MDA reflects downstream aldehydic products of lipid peroxidation, whereas 8-iso-PGF2α is generated through non-enzymatic free radical-catalysed oxidation of arachidonic acid, making it a more chemically specific marker of oxidative membrane injury.

Beyond oxidative mechanisms, systemic inflammation plays a central amplifying role in MASLD pathogenesis. Haematological ratios derived from routine complete blood count—the neutrophil-to-lymphocyte ratio (NLR), monocyte-to-lymphocyte ratio (MLR), and platelet-to-lymphocyte ratio (PLR)—have emerged as cost-effective, reproducible surrogates of the systemic inflammatory state. NLR reflects the balance between neutrophil-driven innate inflammation and lymphocyte-mediated adaptive immunity, and has been validated in multiple studies as a predictor of NASH and significant fibrosis in NAFLD/MASLD patients [13,14]. In T2DM specifically, elevated NLR correlates with poor glycemic control and increased insulin resistance [15], suggesting a pathological synergy between metabolic dysregulation and systemic immune imbalance. MLR reflects activation of the monocyte-macrophage axis; circulating monocytes recruited to the liver differentiate into pro-inflammatory and pro-fibrotic macrophages (monocyte-derived macrophages, MoMFs), playing a central role in the progression from hepatic steatosis to steatohepatitis and fibrosis [16,17]. The PLR—inversely related to platelet count, which declines in advanced fibrosis due to hypersplenism—has demonstrated utility as an indirect marker of hepatic fibrosis severity across multiple liver disease etiologies [18,19].

Non-invasive stratification of hepatic fibrosis risk is a cornerstone of MASLD management. The FIB-4 index—calculated from age, AST, ALT, and platelet count—is the most extensively validated blood-based tool for fibrosis risk assessment and is recommended as a first-line instrument by both EASL and AASLD guidelines [4,20]. Patients with T2DM are specifically identified as a priority population for systematic FIB-4 screening given their elevated risk of advanced fibrosis [4,5]. However, the precise relationships between FIB-4 and the inflammatory haematological ratios—particularly in the context of diabetic MASLD—remain incompletely elucidated.

Despite growing evidence that individually links oxidative stress biomarkers and systemic inflammatory indices to MASLD severity, a simultaneous evaluation of MDA, 8-iso-PGF2α, NLR, MLR, and PLR alongside the FIB-4 score in a cohort of diabetic and non-diabetic MASLD patients has not been comprehensively reported. Furthermore, longitudinal data characterising the temporal dynamics of these biomarkers across successive clinical evaluations in patients with diabetic MASLD remain scarce. The present study aims to address these gaps by: (i) comparing oxidative stress and systemic inflammatory profiles between MASLD patients with and without T2DM; (ii) evaluating correlations between oxidative markers, inflammatory indices, and FIB-4; and (iii) characterising the longitudinal evolution of these biomarkers across three successive clinical timepoints in the diabetic cohort. To the best of our knowledge, this is the first study to simultaneously address these three objectives in a cohort of Romanian patients with MASLD.

## 2. Results

### 2.1. Baseline Characteristics of the Study Population

The study enrolled 110 patients, stratified into two groups: Group 1 (G1, n = 20), comprising patients with MASLD and concurrent Type 2 Diabetes Mellitus (T2DM), and Group 2 (G2, n = 90), comprising non-diabetic patients with MASLD-spectrum liver disease, including patients with advanced fibrosis or cirrhotic-stage hepatic involvement, evaluated cross-sectionally. Complete baseline characteristics and between-group comparisons are presented in Table 1.

A statistically significant difference in sex distribution was observed, with a higher proportion of female patients in G1 (60%) compared to G2 (23%) (Fisher’s exact test, *p* = 0.002). Age was comparable between groups (G1: 59.80 ± 12.76 years vs. G2: 59.50 [51.00–66.00] years; Mann–Whitney U, *p* = 0.579). As anticipated, fasting glucose was markedly elevated in G1 (169.50 [118.00–305.25] mg/dL) relative to G2 (106.00 [95.00–122.00] mg/dL; *p* < 0.001), consistent with the diabetic phenotype. Mean HbA1c in G1 was 8.51 ± 1.97%, reflecting suboptimal glycemic control at the time of enrollment.

With respect to hepatic parameters, AST was significantly higher in G2 (79.00 [52.00–93.00] U/L vs. G1: 40.00 [19.75–66.00] U/L; *p* < 0.001), as were ALT (G2: 44.50 [27.00–68.00] U/L vs. G1: 30.50 [20.00–36.00] U/L; *p* = 0.025), total bilirubin (G2: 3.44 [1.03–5.71] mg/dL vs. G1: 0.58 [0.48–0.77] mg/dL; *p* < 0.001), and the FIB-4 fibrosis score (G2: 14.44 [6.99–50.00] vs. G1: 1.79 [1.28–2.61]; *p* < 0.001). These findings confirm a more advanced degree of hepatic structural injury in the non-diabetic cirrhotic cohort. Haemoglobin was significantly higher in G1 (13.95 [13.02–14.55] g/dL) than in G2 (10.10 [8.30–12.20] g/dL; *p* < 0.001), reflecting the anaemia commonly observed in decompensated cirrhosis. Lymphocyte, monocyte, and neutrophil absolute counts were substantially higher in G1, as G2 patients exhibited marked cytopenia secondary to hypersplenism (all *p* < 0.001).

### 2.2. Oxidative Stress Biomarkers

**Figure 1:**
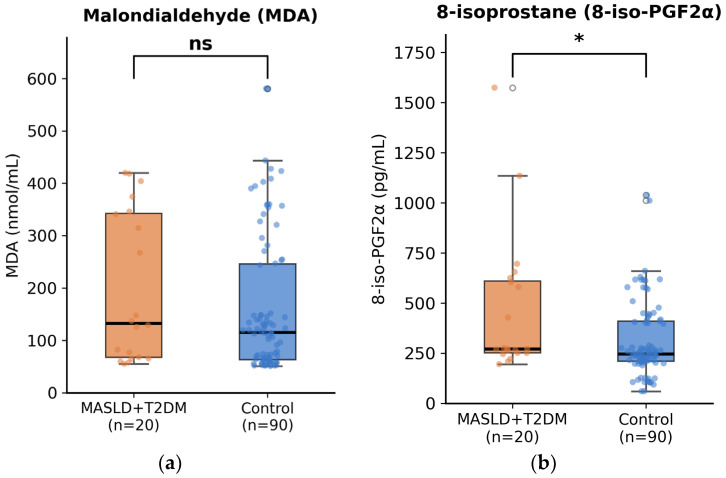
Oxidative stress biomarkers in G1 and G2. (**a**) Malondialdehyde (MDA, nmol/mL); (**b**) 8-isoprostaglandin F2α (8-iso-PGF2α, pg/mL). Boxes show median with IQR; whiskers extend to 1.5 × IQR; individual data points are overlaid. Significance: * *p* < 0.05; Mann–Whitney U test.

Both MDA and 8-iso-PGF2α demonstrated non-normal distributions in both groups (Shapiro–Wilk test, all *p* < 0.05), necessitating non-parametric inference. MDA levels did not differ significantly between G1 (133.01 [67.52–342.37] nmol/mL) and G2 (115.38 [63.21–246.07] nmol/mL) (Mann–Whitney U = 1043.0, *p* = 0.269). However, 8-iso-PGF2α—a highly specific and sensitive marker of non-enzymatic lipid peroxidation—was significantly elevated in G1 compared to G2 (271.93 [251.60–609.56] pg/mL vs. 246.00 [210.14–409.44] pg/mL; Mann–Whitney U = 1219.0, *p* = 0.014), indicating a greater degree of oxidative membrane damage in MASLD patients with concurrent T2DM (Figure 1).

### 2.3. Systemic Inflammatory Indices

**Figure 2:**
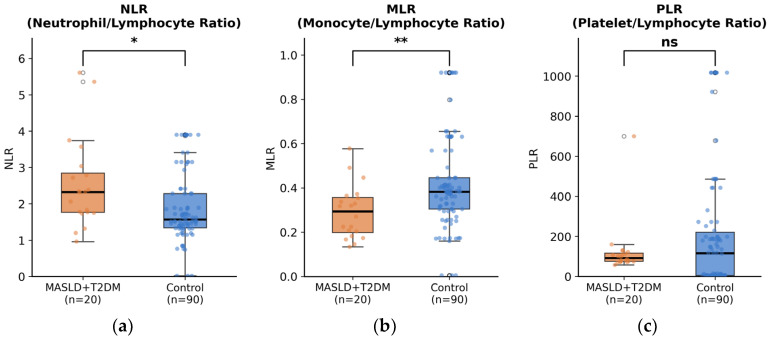
Systemic inflammatory indices in G1 and G2. NLR  =  neutrophil-to-lymphocyte ratio; MLR  =  monocyte-to-lymphocyte ratio; PLR  =  platelet-to-lymphocyte ratio. PLR values clipped at the 95th percentile for visualization. Boxes: median [IQR]; dots: individual values. Mann–Whitney U test. NLR: *p* = 0.016 (*); MLR: *p* = 0.004 (**); PLR: *p* = 0.771.

NLR was significantly higher in G1 compared to G2 (2.32 [1.76–2.84] vs. 1.57 [1.33–2.28]; Mann–Whitney U, *p* = 0.016), indicating a more pronounced neutrophil-dominant systemic inflammatory response in diabetic MASLD (Figure 2a). Conversely, MLR was significantly higher in G2 (0.38 [0.30–0.44] vs. G1: 0.29 ± 0.12; Mann–Whitney U, *p* = 0.004), potentially reflecting the greater monocyte-macrophage hepatic inflammatory activation observed in the cirrhotic non-diabetic cohort (Figure 2b). PLR did not differ significantly between groups (G1: 92.01 [75.19–115.59] vs. G2: 115.90 [3.99–220.76]; Mann–Whitney U, *p* = 0.771) (Figure 2c).

### 2.4. Correlation Analyses

#### 2.4.1. Oxidative Stress Biomarkers and Inflammatory Indices

A significant positive correlation was identified between MLR and MDA in G1 (Spearman r = 0.472, 95% CI [0.037–0.757], *p* = 0.036), suggesting that monocyte-lymphocyte imbalance is associated with greater lipid peroxidation specifically within the diabetic MASLD context. This association was not reproduced in G2 or in the combined cohort, nor did MDA or 8-iso-PGF2α correlate significantly with NLR in any analytical stratum. No significant correlations were detected between either oxidative stress marker and FIB-4, AST, ALT, glucose, or HbA1c (all *p* > 0.05), likely reflecting the limited statistical power conferred by the G1 sample size (n = 20).

#### 2.4.2. Inflammatory Indices and FIB-4

**Figure 3:**
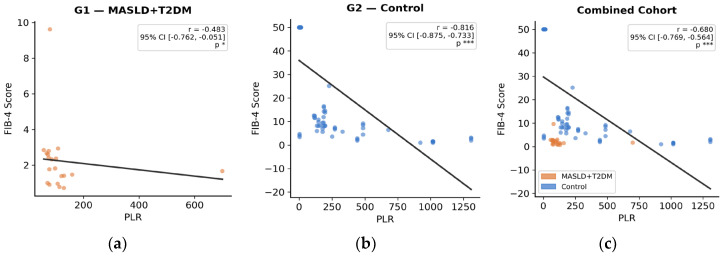
Scatter plots of PLR vs. FIB-4 Score in (**a**) G1—MASLD + T2DM, (**b**) G2—Control, and (**c**) Combined cohort. Lines represent Spearman regression fits. Spearman r, 95% CI, and significance levels are displayed in each panel. Orange dots  =  G1; Blue dots  =  G2; * *p* < 0.05; *** *p* < 0.001.

A strong and consistent negative correlation was observed between PLR and FIB-4 across all analytical strata: in G1 (Spearman r = −0.483, 95% CI [−0.762, −0.051], *p* = 0.031), in G2 (Spearman r = −0.816, 95% CI [−0.875, −0.733], *p* < 0.001), and in the combined cohort (Spearman r = −0.680, 95% CI [−0.769, −0.564], *p* < 0.001) (Figure 3). This inverse relationship is mechanistically coherent with the progressive thrombocytopenia associated with portal hypertension in advanced liver fibrosis, which simultaneously depresses PLR and drives FIB-4 upward through its platelet component. Additionally, NLR showed a statistically significant negative correlation with FIB-4 in the combined cohort (Spearman r = −0.264, 95% CI [−0.430, −0.081], *p* = 0.005). A borderline significant positive association between MLR and AST was observed in the combined cohort (r = 0.188, *p* = 0.050). The complete correlation matrix for all tested pairs is presented in Table 2.

**Figure 4:**
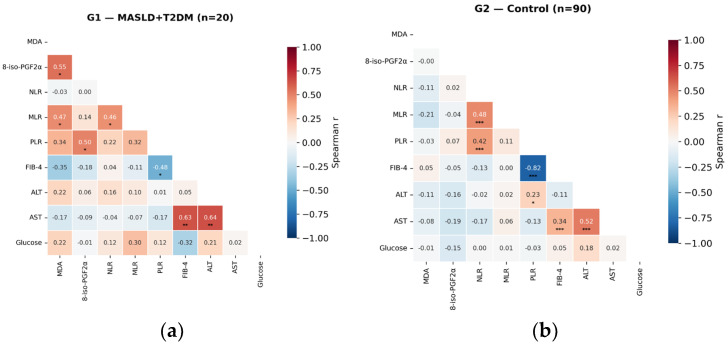
Spearman correlation heatmaps for (**a**) G1—MASLD + T2DM (n = 20) and (**b**) G2—Control (n = 90). Heatmaps are displayed vertically to improve readability. Color scale: dark blue  =  r  =  −1; white  =  r  =  0; dark red  =  r  =  +1. Asterisks: * *p* < 0.05; ** *p* < 0.01; *** *p* < 0.001.

Spearman correlation heatmaps for G1 and G2 are presented in Figure 4, illustrating the overall pattern of associations between all primary study variables.

### 2.5. Longitudinal Analysis of Key Parameters in G1

**Figure 5:**
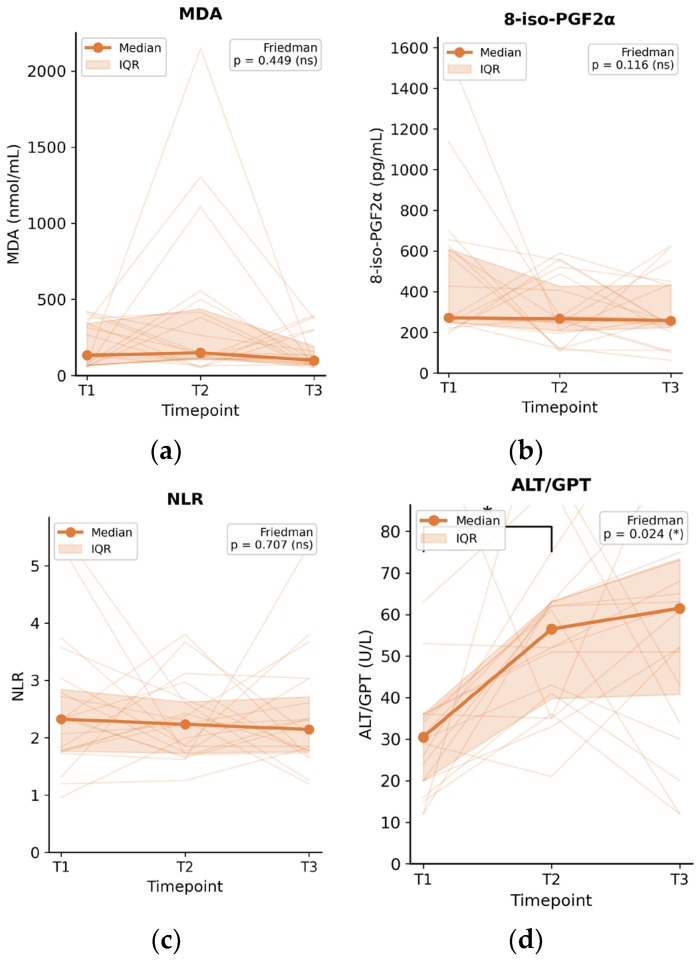
Longitudinal evolution of key biomarkers in MASLD + T2DM patients (G1, n = 20) across T1, T2, T3. Thin lines  =  individual patient trajectories; bold line  =  group median; shaded area  =  IQR. Friedman *p*-values shown. ALT: *p* = 0.024 (*); post hoc Wilcoxon T1 vs. T2 *p*_adj = 0.014 (*). All other parameters: * *p* > 0.05.

Among the 20 G1 patients monitored at three successive clinical timepoints (T1, T2, T3), the Friedman test revealed no statistically significant temporal variation in oxidative stress markers: MDA (χ^2^ = 1.600, *p* = 0.449) (Figure 5a) and 8-iso-PGF2α (χ^2^ = 4.300, *p* = 0.117) (Figure 5b). Similarly, no significant changes were detected in NLR (χ^2^ = 0.692, *p* = 0.707) (Figure 5c), MLR (χ^2^ = 0.179, *p* = 0.914), or FIB-4 (χ^2^ = 1.380, *p* = 0.502), suggesting overall biomarker stability during clinical follow-up (Table 3).

Notably, ALT showed a statistically significant increase across time points (Friedman χ^2^ = 7.462, *p* = 0.024) (Figure 5d). Post hoc Wilcoxon analysis with Bonferroni correction identified a significant elevation from T1 (30.50 [20.00–36.00] U/L) to T2 (56.50 [39.75–63.00] U/L) (*p*_adj = 0.014), while T1 vs. T3 did not reach corrected significance (*p*_adj = 0.109). Glucose and HbA1c showed non-significant upward trends (*p* = 0.143 and *p* = 0.576, respectively).

### 2.6. Multivariate Regression Analysis

#### 2.6.1. Predictors of FIB-4 in G1

Multiple linear regression in G1 identified a model significantly predictive of FIB-4 variance (F = 8.282, *p* = 0.001, R^2^ = 0.688, Adjusted R^2^ = 0.605). ALT (β = −0.078, 95% CI [−0.114, −0.042], *p* < 0.001) and AST (β = 0.072, 95% CI [0.045, 0.099], *p* < 0.001) were the only statistically significant independent predictors. NLR and MLR did not contribute significantly (*p* > 0.05).

#### 2.6.2. Predictors of FIB-4 in G2

In G2 (n = 90), the model explained 32.2% of FIB-4 variance (F = 7.986, *p* < 0.001, R^2^ = 0.322, Adjusted R^2^ = 0.282). ALT (β = −0.405, *p* < 0.001) and AST (β = 0.225, *p* < 0.001) were the sole independent predictors. Age, NLR, and MLR were not significant contributors.

#### 2.6.3. Predictors of MDA in G1

A regression model for MDA in G1 (F = 2.962, *p* = 0.055, R^2^ = 0.441, Adjusted R^2^ = 0.292) identified MLR as the only statistically significant predictor (β = 841.78, 95% CI [203.03, 1480.52], *p* = 0.013), independently of NLR, glucose, and AST. Full regression outputs are presented in Table 4. Given the significant between-group difference in sex distribution, additional sex-adjusted regression analyses were performed in the MASLD + T2DM cohort. After adjustment for sex, ALT and AST remained significant predictors of FIB-4, while MLR remained the only independent predictor of MDA. These findings suggest that the principal observed associations were not primarily driven by sex imbalance between study groups.

To address the significant between-group difference in sex distribution, additional sex-adjusted regression analyses were performed in the MASLD + T2DM cohort. After inclusion of sex as a covariate, the FIB-4 model remained statistically significant (R^2^ = 0.793, adjusted R^2^ = 0.719, F = 10.734, *p* < 0.001). ALT (β = −0.073, *p* < 0.001) and AST (β = 0.070, *p* < 0.001) remained independent predictors of FIB-4. Sex was also independently associated with FIB-4 in this model (β = −1.332, *p* = 0.019).

In the sex-adjusted MDA model, MLR remained the only significant independent predictor of MDA (β = 955.92, 95% CI [266.40, 1645.44], *p* = 0.010), whereas sex was not significantly associated with MDA (β = 66.73, *p* = 0.340). These findings support the robustness of the observed MLR–MDA association after accounting for sex distribution.

## 3. Discussion

The present study examined oxidative stress biomarkers (MDA, 8-iso-PGF2α), systemic inflammatory indices (NLR, MLR, PLR), and the FIB-4 fibrosis score in a cohort of 110 patients with MASLD stratified by the presence or absence of T2DM, with an additional longitudinal analysis of 20 diabetic patients followed at three successive timepoints. Our results reveal a distinct inflammatory and oxidative phenotype in diabetic MASLD, characterised by significantly elevated 8-iso-PGF2α and NLR, alongside novel associations between MLR and lipid peroxidation (MDA) and between PLR and the risk of hepatic fibrosis.

### 3.1. Oxidative Stress Biomarkers: 8-iso-PGF2α as a Discriminating Marker in Diabetic MASLD

The finding that 8-iso-PGF2α was significantly elevated in G1 (MASLD + T2DM) compared to G2 (271.93 vs. 246.00 pg/mL; *p* = 0.014) is consistent with the established mechanistic link between chronic hyperglycemia and enhanced non-enzymatic arachidonic acid peroxidation. Hyperglycemia drives mitochondrial superoxide overproduction, activating multiple oxidative pathways—including the polyol pathway, AGE formation, PKC activation, and NADPH oxidase—that collectively amplify the ROS burden [8]. The preferential elevation of 8-iso-PGF2α over MDA in our diabetic group aligns with the superior biochemical specificity of isoprostanes as non-enzymatic peroxidation products, whereas MDA can also arise from enzymatic pathways and is thus subject to greater biological variability [12,21]. Our findings are consistent with prior evidence demonstrating enhanced urinary 8-iso-PGF2α excretion in T2DM patients that correlates with impaired glycemic control, a relationship formally established in mechanistic studies [11].

In contrast, MDA did not differ significantly between groups (133.01 vs. 115.38 nmol/mL; *p* = 0.269). This apparent dissociation between MDA and 8-iso-PGF2α has been reported in other clinical settings [10] and may reflect the more complex metabolic fate of MDA—including its reactivity with proteins and nucleic acids, as well as its rapid tissue clearance—compared with the more chemically stable isoprostane fraction [12]. Serum MDA has nonetheless been associated with NAFLD severity in a dose–response manner in large cross-sectional cohorts [22], and the absence of a between-group difference in our study may be attributable to the limited sample size of G1 (n = 20), which constrains the statistical power to detect moderate effect sizes.

### 3.2. Systemic Inflammatory Indices: NLR, MLR, and Their Divergent Profiles

NLR was significantly elevated in the diabetic MASLD group (2.32 vs. 1.57; *p* = 0.016), consistent with the established role of T2DM as a driver of systemic, neutrophil-dominant chronic low-grade inflammation. This finding aligns with published meta-analytic evidence showing that NLR is positively correlated with glycemic indices in T2DM populations, with higher NLR values observed in patients with poor HbA1c control [15]. In the MASLD context, a systematic review and meta-analysis of 13 studies confirmed that NLR is elevated in NAFLD patients with significant NASH (SMD = 0.97) and advanced fibrosis (SMD = 1.59) [13], and a recent population-based cohort study of 16,859 MASLD patients showed that NLR independently predicts all-cause and cardiovascular mortality [14]. Our G1 median NLR of 2.32 is consistent with published values for diabetic NAFLD/MASLD populations [23] and supports the conclusion that T2DM amplifies the neutrophilic inflammatory response in hepatic steatosis.

The finding that MLR was significantly higher in G2 (0.38 vs. 0.29; *p* = 0.004) warrants careful mechanistic consideration. G2 patients presented with more advanced hepatic structural damage—as evidenced by higher AST, total bilirubin, and FIB-4—consistent with a cirrhotic phenotype with portal hypertension. In advanced liver disease, circulating monocyte counts may be elevated due to ongoing hepatic monocyte recruitment, as resident Kupffer cells are progressively depleted and replaced by monocyte-derived macrophages (MoMFs) [16,17]. This activation of the monocyte-macrophage axis, reflected by higher MLR in our cirrhotic non-diabetic cohort, is consistent with published evidence that MoMF infiltration is particularly pronounced in advanced MASH and cirrhosis [17]. While the higher MLR in G2 might appear counterintuitive—as diabetes is the inflammatory condition—it likely reflects the more advanced stage of liver disease in G2, rather than a lower inflammatory burden in G1.

PLR did not differ significantly between groups (92.01 vs. 115.90; *p* = 0.771). The high variability of PLR in G2 (IQR [3.99–220.76]) likely reflects the wide heterogeneity of platelet counts in cirrhotic patients, ranging from severe thrombocytopenia (due to hypersplenism) to near-normal counts, depending on disease stage and medication [18,19].

### 3.3. Correlation Between PLR and FIB-4: A Consistent Inverse Association

The strong and consistent inverse correlation between PLR and FIB-4 observed across all analytical strata—G1 (r = −0.483, *p* = 0.031), G2 (r = −0.816, *p* < 0.001), and the combined cohort (r = −0.680, *p* < 0.001)—constitutes one of the most clinically meaningful findings of this study. This relationship is mechanistically coherent: hepatic fibrosis and portal hypertension lead to splenic sequestration and reduced thrombopoietin-mediated platelet production, thereby progressively decreasing platelet counts [24]. Since both PLR (numerator: platelets) and FIB-4 (denominator: platelets) are mathematically coupled through their platelet components, an inverse relationship is expected and has been consistently reported in liver disease populations [18,19]. A recent nationwide cross-sectional study using NHANES data demonstrated that PLR is inversely associated with the risk of cirrhosis in patients with NAFLD [18], while a European study confirmed PLR as a predictor of advanced fibrosis across multiple hepatic conditions [19]. The particularly strong correlation in G2 (r = −0.816) reflects the wide spectrum of fibrosis severity in the cirrhotic cohort, providing greater statistical power to detect this association.

### 3.4. MLR as an Independent Predictor of MDA: A Novel Mechanistic Link

The identification of MLR as the only significant predictor of MDA in the G1 regression model (β = 841.78, *p* = 0.013) represents a potentially novel finding with mechanistic implications. Monocytes are significant sources of ROS via NADPH oxidase-mediated pathways and can directly contribute to lipid peroxidation in inflamed tissues [16]. In the context of diabetic MASLD, where circulating monocytes are primed toward pro-inflammatory (M1-like) polarisation by hyperglycemia and advanced glycation end-products (AGEs), their capacity to generate superoxide and hydrogen peroxide—thereby driving MDA formation—may be amplified [8]. This Spearman correlation (r = 0.472, *p* = 0.036) and regression coefficient are consistent with a monocyte-mediated peroxidation pathway in diabetic hepatic disease, although confirmatory mechanistic studies are needed to establish causality.

### 3.5. Longitudinal Findings: Stability of Oxidative and Inflammatory Markers, with Notable ALT Elevation

The longitudinal analysis of 20 G1 patients across three clinical time points revealed no statistically significant changes in oxidative stress markers (MDA, 8-iso-PGF2α), inflammatory indices (NLR, MLR), or FIB-4 over the observation period (all Friedman *p* > 0.05). This relative stability may reflect either genuine biomarker homeostasis—potentially mediated by antidiabetic or hepatoprotective medications—or insufficient follow-up duration to capture meaningful biological changes. The limited sample size (n = 20) and unknown inter-measurement intervals also constrain the interpretation of longitudinal trends.

The significant longitudinal increase in ALT (Friedman χ^2^ = 7.462, *p* = 0.024), with a significant T1 → T2 rise (30.50 to 56.50 U/L; Wilcoxon Bonferroni-corrected *p* = 0.014), is notable. Progressive ALT elevation without parallel changes in oxidative or inflammatory indices suggests a pattern of subclinical hepatocellular injury that may be driven by mechanisms not captured by the measured biomarkers—such as lipotoxicity, endoplasmic reticulum stress, or medication-related hepatotoxicity [25]. The absence of synchronous elevation in FIB-4 (*p* = 0.502) may indicate that the observed increase in ALT reflects transient hepatocellular damage rather than progressive fibrogenesis over the study period, though longer follow-up would be required to confirm this [4].

### 3.6. Regression Models for FIB-4: Dominance of Liver Enzymes over Inflammatory Indices

In both groups, multiple linear regression identified ALT and AST as the only independent predictors of FIB-4, while NLR, MLR, and age did not contribute significantly. This finding is expected mathematically—FIB-4 is defined as (Age × AST)/(Platelets × √ALT), meaning that ALT and AST are integral components of the score and will naturally emerge as dominant predictors in any regression model using FIB-4 as the dependent variable. The high R^2^ in G1 (0.688), despite the small sample size, suggests a good model fit, though the narrow confidence intervals for β may be unstable at n = 20 [20]. The G2 model explained 32.2% of the variance in FIB-4 (R^2^ = 0.322), consistent with the mathematical contribution of liver enzymes after accounting for platelet and age effects.

### 3.7. Study Limitations

Several limitations of the present study merit acknowledgement. First, the small sample size of G1 (n = 20) significantly limits statistical power for multivariate analyses, correlation detection, and longitudinal trend assessment. Based on a priori power calculations, 50–80 patients per group would be recommended for reliable Spearman correlation analyses at the observed effect sizes. Second, the inter-measurement intervals between T1, T2, and T3 were not standardised and were unavailable for the present analysis; this information is essential for interpreting longitudinal changes. Third, FIB-4 in G2 was estimated from formula components rather than extracted directly from clinical records, which may introduce systematic bias. Fourth, although both groups were matched by age, significant differences in sex distribution (60% female in G1 vs. 23% in G2; *p* = 0.002) and baseline hepatic disease severity were observed, limiting comparability between groups and constituting a potential confounding factor. To partially address this issue, additional sex-adjusted regression analyses were performed, confirming the persistence of the principal observed associations. Fifth, clinical covariates with known impact on oxidative stress and inflammation—including BMI, smoking status, alcohol consumption, antidiabetic medication (particularly metformin and GLP-1 agonists), hepatotropic therapy, and statin use—were not systematically available in a harmonised format within the source database and therefore could not be incorporated into the present analyses [4,20]. Additionally, the non-diabetic comparator group did not represent a healthy control population, but rather a heterogeneous MASLD-spectrum cohort that included patients with more advanced hepatic disease. Consequently, between-group comparisons should primarily be interpreted as reflecting differences between diabetic and non-diabetic MASLD phenotypes across varying stages of liver disease severity. Future studies with larger cohorts, standardised follow-up intervals, complete covariate adjustment, and histological validation are needed to confirm and expand the findings of the present investigation.

## 4. Materials and Methods

### 4.1. Study Design and Population

This was a single-centre observational study with a combined cross-sectional and longitudinal design, carried out between October 2025 and March 2026 at the Diabetes Clinic of the Craiova County Emergency Hospital, Romania. A total of 110 adult patients with confirmed metabolic dysfunction-associated steatotic liver disease (MASLD) were enrolled and allocated into two groups based on the concurrent presence or absence of type 2 diabetes mellitus (T2DM).

The MASLD diagnosis was established following the 2023 multisociety Delphi consensus statement [1], which requires evidence of hepatic steatosis on imaging together with at least one cardiometabolic risk criterion from the following five: (i) body mass index (BMI) ≥ 25 kg/m^2^ (≥23 kg/m^2^ in Asian populations) or waist circumference exceeding 94 cm in men and 80 cm in women; (ii) fasting plasma glucose ≥ 100 mg/dL, post-load glucose ≥ 140 mg/dL at two hours, HbA1c ≥ 5.7%, or existing T2DM treatment; (iii) blood pressure ≥ 130/85 mmHg or use of antihypertensive medication; (iv) serum triglycerides ≥150 mg/dL or lipid-lowering therapy; or (v) HDL cholesterol below 40 mg/dL in men or 50 mg/dL in women, or corresponding pharmacological management [1]. T2DM was defined per American Diabetes Association standards: HbA1c ≥ 6.5% or fasting glycemia ≥ 126 mg/dL on two separate determinations, or classic hyperglycemia symptoms accompanied by a random glucose reading ≥ 200 mg/dL.

The study population was organised as follows:

- Group 1 (G1—Cases): 20 patients with MASLD and concurrent T2DM, prospectively followed at three successive clinical evaluations (T1, T2, T3), forming the longitudinal arm of the study.
- Group 2 (G2—Controls): 90 patients presenting with MASLD or other advanced hepatic pathology in the absence of T2DM, enrolled at a single cross-sectional evaluation.

The longitudinal component was restricted to the MASLD + T2DM cohort because these patients were prospectively monitored through scheduled diabetology follow-up visits, enabling repeated clinical and biochemical assessments at successive time points. In contrast, the non-diabetic cohort underwent a single cross-sectional clinical evaluation, and standardised repeated follow-up assessments were not available for these participants.

Patients were considered eligible if they were at least 18 years of age, carried a confirmed MASLD diagnosis as defined above, and had a complete set of clinical and laboratory data available at the time of inclusion. Patients were excluded in the presence of any of the following conditions: alternative, well-established causes of chronic liver disease (including chronic hepatitis B or C virus infection, autoimmune hepatitis, primary biliary cholangitis, primary sclerosing cholangitis, hereditary hemochromatosis, or Wilson’s disease); alcohol intake surpassing the thresholds specified in the MASLD consensus definition (>20 g/day for women and >30 g/day for men) [1]; active oncological or hematological disease; receipt of hepatotoxic medications (systemic corticosteroids, amiodarone, tamoxifen, or valproate) within the six months prior to enrollment; an intercurrent acute infection or systemic inflammatory episode at the time of sample collection; end-stage renal disease necessitating dialysis; or missing data rendering the patient’s dataset incomplete.

Information regarding chronic antidiabetic, hepatotropic, and lipid-lowering therapies was not systematically available in a harmonised format within the source database and, therefore, could not be reliably incorporated into the inclusion/exclusion criteria or multivariable analyses.

The study protocol was conducted in accordance with the ethical principles set forth in the Declaration of Helsinki (2013 revision) and received approval from the Ethics Committee of the University of Medicine and Pharmacy of Craiova (protocol code 225/27 August 2024). All participants provided written informed consent prior to their inclusion in the study.

### 4.2. Sample Size Calculation

An a priori power analysis was performed using G*Power, version 3.1.9.7 (Heinrich-Heine-Universität Düsseldorf, Düsseldorf, Germany). Drawing on published evidence of NLR differences between MASLD populations stratified by T2DM status [13,21], a medium-to-large effect size of d = 0.65 was assumed for the primary between-group comparison. At a two-sided alpha level of 0.05 and a target statistical power of 0.80, with an enrollment ratio of 1:4.5 (G1:G2), the minimum sample sizes were 18 for G1 and 80 for G2. Final recruitment targets were set at n = 20 and n = 90, respectively, to maintain an adequate margin of safety above these minima.

### 4.3. Clinical and Laboratory Data Collection

Demographic information (age, sex) was recorded at the time of enrollment. Blood samples were drawn from the antecubital vein in the morning, following a minimum fasting period of 10 h. All biochemical and hematological determinations were completed within four hours of venipuncture at the accredited Clinical Laboratory of the Craiova County Emergency Hospital, employing validated automated analytical platforms operated under continuous internal quality-control procedures. Because the present analysis was conducted on an anonymized research database derived from routine clinical records, the exact analyzer models were not consistently available for all participants.

Laboratory investigations encompassed the following domains. Hepatic function was evaluated through alanine aminotransferase (ALT, U/L), aspartate aminotransferase (AST, U/L), gamma-glutamyl transferase (GGT, U/L), alkaline phosphatase (ALP, U/L), direct bilirubin (BD, mg/dL), and total bilirubin (BT, mg/dL). Metabolic and renal parameters included fasting plasma glucose (mg/dL), glycated hemoglobin (HbA1c, %), serum urea (mg/dL), creatinine (mg/dL), sodium (Na, mmol/L), and potassium (K, mmol/L). Hematological indices encompassed total leukocyte count (/µL), absolute neutrophil, lymphocyte, and monocyte counts (×10^9^/L), platelet count (/µL), hemoglobin (g/dL), hematocrit (%), red cell distribution width (RDW, %), and mean corpuscular volume (MCV, fL). Additionally, serum amylase (U/L) was measured as part of the metabolic and pancreatic assessment.

Hepatic steatosis was confirmed and graded by abdominal ultrasonography, performed by a trained physician according to a standardised imaging protocol using routine hospital ultrasound equipment. Because imaging data were extracted from anonymized clinical records, the exact ultrasound device models and probe specifications were not consistently available for all participants. In Group 1 participants, glycemic control was reassessed at each follow-up visit by measuring HbA1c.

### 4.4. Derived Indices and Fibrosis Assessment

Systemic inflammatory indices were calculated from the complete blood count. In Group 1, only leukocyte differential percentages were available; absolute counts were therefore derived prior to index computation according to:

- Absolute count (×10^9^/L) = [Total leukocytes (/µL) × Differential (%)]/100,000
- This step ensured that all inflammatory ratio calculations were performed using values expressed in the same unit (×10^9^/L) as those directly reported by the haematology analyser in Group 2. Three haematology-based inflammatory ratios were then derived:
- NLR = Absolute neutrophil count/Absolute lymphocyte count
- MLR = Absolute monocyte count/Absolute lymphocyte count
- PLR = Absolute platelet count (×10^9^/L)/Absolute lymphocyte count
- For PLR computation, the platelet count in/µL was converted to ×10^9^/L by dividing by 1000 prior to the ratio calculation.

Hepatic fibrosis risk was quantified using the FIB-4 index [20], derived as:

- FIB-4 = [Age (years) × AST (U/L)]/[Platelet count (×10^9^/L) × √ALT (U/L)]

Direct FIB-4 readings from clinical records were used for Group 1 patients; for Group 2, the index was recalculated from individual laboratory components. Risk stratification followed the EASL-endorsed cut-off values: FIB-4 below 1.30 was interpreted as low fibrosis risk; values between 1.30 and 2.67 as indeterminate, warranting further imaging-based assessment; and values exceeding 2.67 as indicative of high fibrosis risk, consistent with advanced hepatic fibrosis [4,20].

### 4.5. Quantification of Oxidative Stress Biomarkers

Two circulating markers of lipid peroxidation were selected to characterise systemic oxidative stress. Malondialdehyde (MDA), expressed in nmol/mL, is a reactive aldehyde generated as a downstream product of polyunsaturated fatty acid peroxidation and represents one of the most broadly employed proxies of oxidative lipid damage in clinical investigation [10]. 8-epi-prostaglandin F2α (8-epi-PGF2α), expressed in pg/mL, is a stable isoprostane produced exclusively through non-enzymatic, free radical-catalysed oxidation of arachidonic acid esterified in membrane phospholipids; its formation is independent of cyclooxygenase activity, rendering it a chemically specific and biologically reliable indicator of in vivo oxidative stress burden [12,22].

Serum concentrations of both analytes were determined at the Immunology Laboratory of the University of Medicine and Pharmacy of Craiova using a competitive enzyme-linked immunosorbent assay (ELISA) format with commercially available kits from Elabscience (Houston, TX, USA): Catalog No. E-EL-0060 for MDA (analytical sensitivity: 18.75 ng/mL; quantification range: 31.25–2000 ng/mL) and Catalog No. E-EL-0041 for 8-epi-PGF2α (analytical sensitivity: 9.38 pg/mL; quantification range: 15.63–1000 pg/mL). Intra-assay and inter-assay precision, expressed as coefficients of variation (CV%), ranged from 4.07 to 6.86% and from 7.09 to 7.77%, respectively, as specified in the manufacturer’s lot-specific validation documentation.

The competitive assay format is based on the competition between the analyte present in patient samples and a fixed, plate-bound antigen for a limited pool of biotinylated detection antibodies. Bound detection antibody is subsequently revealed by horseradish peroxidase—conjugated avidin, followed by colorimetric development with 3,3′,5,5′-tetramethylbenzidine substrate. The reaction is terminated by addition of a stop solution, and optical density is measured at 450 ± 2 nm using the Asys Expert Plus UV G020 150 Microplate Reader (ASYS Hitech GmbH, Eugendorf, Austria). Because signal intensity is inversely proportional to analyte concentration, sample values are interpolated from a calibration curve constructed from optical density readings of serial calibrators. Antibody cross-reactivity with structurally related compounds was assessed by the manufacturer and reported as negligible for both assays (cross-reactivity data accessible at https://789.bio/ea/OC4Om5 and https://789.bio/ea/GG8Gy9, both accessed on 21 September 2025).

To safeguard pre-analytical integrity, blood samples were processed without delay: serum was obtained by centrifugation within 30 min of collection, immediately portioned into single-use cryovials, and preserved at −80 °C until analysis. Each aliquot was subjected to a single freeze–thaw cycle. Manufacturer-supplied diluents incorporating stabilizing agents were used throughout to reduce background interference. Spike-and-recovery and parallelism experiments were not performed in-house; accordingly, although the manufacturer’s documentation confirms high analyte specificity, the possibility of residual matrix interference cannot be categorically excluded, and results should be interpreted with this caveat in mind.

### 4.6. Statistical Analysis

Primary data were first organised and categorised for statistical analysis using Microsoft Excel 2020 (Microsoft Corporation, Redmond, WA, USA). The data were then categorised into subgroups based on the required analysis type, and derived variables (NLR, MLR, PLR, FIB-4, absolute leukocyte differential counts) were computed prior to database export. Two structured datasets were prepared for analysis: a cross-sectional dataset (n = 110; comprising G1 at T1 and G2 in its entirety) intended for between-group comparisons, correlation analyses, and regression modelling; and a longitudinal dataset (n = 60; 20 patients × 3 timepoints) reserved for repeated-measures analyses. IBM SPSS Statistics, version 26.0 (IBM Corp., Armonk, NY, USA) was used for all inferential analyses. All graphs and figures were produced using GraphPad Prism, version 11 (GraphPad Software, San Diego, CA, USA).

Normality of continuous variables was assessed using the Shapiro–Wilk test, supplemented by scatter plots. For samples with more than 50 observations (Group 2 and the combined cohort), the Kolmogorov–Smirnov test was also applied. Continuous variables are expressed as mean ± standard deviation (SD) for suggestive comparisons and easier clinical understanding, and as median values with interquartile range [Q1–Q3] in statistical comparisons for series without a normal distribution. Nominal data and ordinal variables are presented as absolute and relative frequencies (n, %).

For data that were normally distributed in both groups, between-group differences were assessed using the independent-samples t-test. For data that did not follow a normal distribution in at least one group, the Mann–Whitney U test was applied; the effect size, denoted as r, was computed as the ratio between the absolute value of the standardised test statistic z and the square root of the number of observations (r = |z|/√N). Associations between nominal variables were tested using the Chi-square test, or Fisher’s exact test when expected cell counts fell below five; the magnitude of the categorical association was determined as the ratio between the square root of the chi-square value and the square root of the sample size (w = √(χ^2^/N)). Statistical significance was assessed using an alpha threshold of 5%; *p*-values < 0.05 were considered significant, and the confidence level was set at 95%.

Bivariate correlations between oxidative stress markers, inflammatory indices, FIB-4, and clinically relevant metabolic parameters were assessed using Pearson’s correlation coefficient when both variables met normality criteria, or Spearman’s rank correlation coefficient (ρ) otherwise. Correlation analyses were performed separately within each study group and across the combined cohort. The 95% confidence intervals for correlation coefficients were estimated via the Fisher z-transformation. Association strength was classified as weak (|r| or |ρ| < 0.30), moderate (0.30–0.59), or strong (≥0.60).

Within-subject temporal changes across the three study time points (T1, T2, T3) in Group 1 were examined using the Friedman test, a nonparametric procedure appropriate for repeated measurements from the same subjects. Where the omnibus test reached significance, post hoc pairwise differences were explored with the Wilcoxon signed-rank test, and resulting *p*-values were corrected for multiplicity using Bonferroni’s method (×3 for the three pairwise comparisons: T1 vs. T2, T1 vs. T3, and T2 vs. T3); a corrected *p*-value ≤ 0.017 was required for post hoc significance.

Independent predictors of FIB-4 and oxidative stress markers were identified using multiple linear regression with the Enter (simultaneous) method, with candidate predictors selected based on biological plausibility and univariate correlation results. The quality of each model was characterized by R^2^ and adjusted R^2^. Before interpreting regression outputs, four diagnostic checks were carried out: multicollinearity was evaluated using the variance inflation factor (VIF; threshold VIF < 5); normality of residuals was tested by the Shapiro–Wilk procedure applied to standardized residuals; homoscedasticity was judged from the scatter plot of standardized predicted values versus standardized residuals (ZPRED vs. ZRESID); and undue influence was quantified via Cook’s distance (threshold D < 1). Model results are reported as unstandardized regression coefficients (β) with 95% confidence intervals, t-statistics, and *p*-values.

Box-and-whisker plots display the median and interquartile range, with Tukey-style whiskers and individual data points overlaid. Scatter plots for correlation analyses include the fitted regression line and the 95% confidence band. Longitudinal trajectories are illustrated as median lines with IQR shading and individual patient-level curves rendered as light background elements to convey within-group variability.

## 5. Conclusions

The present study demonstrates that diabetic MASLD (MASLD + T2DM) is characterised by a distinct inflammatory and oxidative stress phenotype, evidenced by significantly elevated 8-iso-PGF2α—a specific marker of non-enzymatic lipid peroxidation—and higher NLR, reflecting greater systemic neutrophil-driven inflammation compared to non-diabetic MASLD/hepatic disease patients. A robust and consistent inverse correlation between PLR and the FIB-4 fibrosis score was identified across both groups and the combined cohort, mechanistically anchored in platelet-mediated fibrosis progression. Notably, MLR emerged as an independent predictor of MDA in diabetic MASLD, suggesting a monocyte-driven oxidative stress pathway that merits further mechanistic investigation. Importantly, this association remained significant after adjustment for sex, supporting the robustness of the observed relationship despite the unequal sex distribution between study groups. Longitudinal analysis demonstrated stability of oxidative and inflammatory biomarkers over the observation period, with a clinically relevant progressive increase in ALT suggesting subclinical hepatocellular injury trajectories in the diabetic cohort. These findings support the utility of routinely available haematological ratios and lipid peroxidation biomarkers for phenotypic characterisation of MASLD in the context of T2DM and highlight the need for prospective longitudinal studies with larger, histologically validated cohorts.

## Figures and Tables

**Table 1:** Baseline characteristics of the study groups.

Parameter	MASLD + T2DM (G1, n = 20)	Control (G2, n = 90)	*p*-Value
DEMOGRAPHICS and GLYCEMIC CONTROL			
Sex—Female, n (%)	12 (60%)	21 (23%)	0.002 **^,a^
Age (years)	59.80 ± 12.76	59.50 [51.00–66.00]	0.579 ^b^
Fasting Glucose (mg/dL)	169.50 [118.00–305.25]	106.00 [95.00–122.00]	<0.001 ***^,b^
HbA1c (%)	8.51 ± 1.97	-	-
LIVER FUNCTION PARAMETERS			
ALT/GPT (U/L)	30.50 [20.00–36.00]	44.50 [27.00–68.00]	0.025 *^,b^
AST/GOT (U/L)	40.00 [19.75–66.00]	79.00 [52.00–93.00]	<0.001 ***^,b^
GGT (U/L)	40.50 [30.00–119.25]	N/A	—
Alkaline Phosphatase (U/L)	96.80 ± 28.29	111.50 [86.87–133.00]	0.061
Direct Bilirubin (mg/dL)	0.20 [0.15–0.33]	2.10 [0.47–3.45]	<0.001 ***^,b^
Total Bilirubin (mg/dL)	0.58 [0.48–0.77]	3.44 [1.03–5.71]	<0.001 ***^,b^
FIB-4 Score	1.79 [1.28–2.61]	14.44 [6.99–50.00]	<0.001 ***^,b^
OXIDATIVE STRESS BIOMARKERS			
MDA (nmol/mL)	133.01 [67.52–342.37]	115.38 [63.21–246.07]	0.269
8-iso-PGF2α (pg/mL)	271.93 [251.60–609.56]	246.00 [210.14–409.44]	0.014 *^,b^
SYSTEMIC INFLAMMATORY INDICES			
NLR	2.32 [1.76–2.84]	1.57 [1.33–2.28]	0.016 *^,b^
MLR	0.29 ± 0.12	0.38 [0.30–0.44]	0.004 **^,b^
PLR	92.01 [75.19–115.59]	115.90 [3.99–220.76]	0.771
HEMATOLOGICAL PARAMETERS			
Hemoglobin (g/dL)	13.95 [13.02–14.55]	10.10 [8.30–12.20]	<0.001 ***^,b^
Lymphocytes (×10^9^/L)	2.38 [2.04–2.88]	0.32 [0.25–0.36]	<0.001 ***^,b^
Monocytes (×10^9^/L)	0.60 [0.52–0.74]	0.11 [0.11–0.14]	<0.001 ***^,b^
Neutrophils (×10^9^/L)	5.34 [4.47–5.87]	0.53 [0.47–0.55]	<0.001 ***^,b^
Platelets (/µL)	224,000 [178,000–254,000]	56,000 [1518–68,000]	<0.001 ***^,b^
Potassium (mmol/L)	4.40 [4.17–4.70]	4.09 ± 0.66	0.022 *^,b^
Sodium (mmol/L)	136.50 [135.00–138.25]	136.00 [131.00–141.00]	0.473

* Values expressed as Mean ± SD (normally distributed variables) or Median [IQR] (non-normally distributed). Normality assessed by the Shapiro–Wilk test (n ≤ 50) or the Kolmogorov–Smirnov test (n > 50). Statistical tests: ^a^ Fisher’s exact test; ^b^ Mann–Whitney U test. FIB-4 in G2 estimated as (Age × AST)/(Platelets × √ALT). Lymphocyte, monocyte, and neutrophil counts in G1 were converted from differential percentages to absolute values (×10^9^/L = Leukocytes × %/100,000). Abbreviations: MDA, malondialdehyde; 8-iso-PGF2α, 8-isoprostaglandin F2α; NLR, neutrophil-to-lymphocyte ratio; MLR, monocyte-to-lymphocyte ratio; PLR, platelet-to-lymphocyte ratio; FIB-4, Fibrosis-4 index; HbA1c, glycated hemoglobin. Significance: * *p* < 0.05; ** *p* < 0.01; *** *p* < 0.001.

**Table 2:** Spearman correlation analyses between oxidative stress biomarkers, inflammatory indices, and FIB-4 score.

Parameter	Group	n	r (Spearman)	95% CI	*p*-Value
Oxidative Stress Markers					
MDA vs. MLR	G1	20	0.472	[0.037, 0.757]	0.036 *
MDA vs. MLR	Combined	110	−0.144	[−0.323, 0.046]	0.134
MDA vs. FIB-4	G1	20	−0.350	[−0.679, 0.074]	0.130
MDA vs. NLR	G1	20	−0.026	[−0.458, 0.413]	0.915
8-iso-PGF2α vs. FIB-4	G1	20	−0.177	[−0.581, 0.279]	0.455
8-iso-PGF2α vs. NLR	G1	20	0.002	[−0.440, 0.443]	0.995
Inflammatory Indices vs. FIB-4					
NLR vs. FIB-4	G1	20	−0.158	[−0.570, 0.301]	0.505
NLR vs. FIB-4	Combined	110	−0.264	[−0.430, −0.081]	0.005 **
MLR vs. FIB-4	G1	20	−0.099	[−0.528, 0.363]	0.675
PLR vs. FIB-4	G1	20	−0.483	[−0.762, −0.051]	0.031 *
PLR vs. FIB-4	G2	90	−0.816	[−0.875, −0.733]	<0.001 ***
PLR vs. FIB-4	Combined	110	−0.680	[−0.769, −0.564]	<0.001 ***
Inflammatory Indices vs. Liver Enzymes					
NLR vs. AST	Combined	110	−0.207	[−0.380, −0.021]	0.030 *
MLR vs. AST	Combined	110	0.188	[0.000, 0.362]	0.050 *

* All correlations performed using Spearman’s rank coefficient. 95% CI estimated by Fisher’s z-transformation. Results grouped by analytical theme. Abbreviations: MDA, malondialdehyde; 8-iso-PGF2α, 8-isoprostaglandin F2α; NLR, neutrophil-to-lymphocyte ratio; MLR, monocyte-to-lymphocyte ratio; PLR, platelet-to-lymphocyte ratio; FIB-4, Fibrosis-4 index. Significance: * *p* < 0.05; ** *p* < 0.01; *** *p* < 0.001.

**Table 3:** Longitudinal evolution of key parameters in G1 patients (n = 20) across three timepoints.

Parameter	n	T1 Median [IQR]	T2 Median [IQR]	T3 Median [IQR]	Friedman χ^2^	*p*-Value
MDA (nmol/mL)	20	133.01 [67.52–342.37]	149.64 [110.48–437.50]	99.76 [67.65–192.98]	1.600	0.449
8-iso-PGF2α (pg/mL)	20	271.93 [251.60–609.56]	267.13 [207.17–426.94]	257.81 [224.21–430.89]	4.300	0.117
FIB-4 Score	18	2.06 [1.38–2.64]	2.06 [1.73–2.60]	2.42 [1.38–3.05]	1.380	0.502
NLR	20	2.32 [1.76–2.84]	2.23 [1.73–2.62]	2.14 [1.76–2.71]	0.692	0.707
MLR	20	0.29 [0.20–0.36]	0.29 [0.19–0.33]	0.22 [0.19–0.35]	0.179	0.914
ALT/GPT (U/L) †	20	30.50 [20.00–36.00]	56.50 [39.75–63.00]	61.50 [40.75–73.50]	7.462	0.024 *
AST/GOT (U/L)	20	40.00 [19.75–66.00]	53.50 [42.50–68.50]	55.50 [46.00–91.25]	1.584	0.453
Glucose (mg/dL)	20	169.50 [118.00–305.25]	192.00 [162.50–248.75]	194.00 [157.75–289.00]	3.895	0.143
HbA1c (%)	20	8.22 [7.03–9.80]	8.85 [8.43–9.70]	9.39 [8.56–10.43]	1.103	0.576

* Friedman test applied as non-parametric repeated-measures analysis. Post hoc: Wilcoxon signed-rank test with Bonferroni correction (α  =  0.05/3  =  0.017). Values: Median [IQR]. † Post hoc for ALT: T1 vs. T2 *p*_adj = 0.014 (*); T1 vs. T3 *p*_adj = 0.109; T2 vs. T3 *p*_adj = 1.000. Significance: * *p* < 0.05.

**Table 4:** Multiple linear regression: independent predictors of FIB-4 score and MDA.

Predictor	β	SE	t	95% CI	*p*-Value
Model 1—G1: FIB-4 (n = 20, R^2^ = 0.688, Adj.R^2^ = 0.605, F = 8.282, *p* = 0.001)					
Intercept	0.787	0.802	0.982	[−0.922, 2.497]	0.341
NLR	−0.380	0.290	−1.313	[−0.997, 0.237]	0.209
MLR	6.395	3.101	2.062	[−0.214, 13.004]	0.057
ALT/GPT	−0.078	0.017	−4.623	[−0.114, −0.042]	<0.001 ***
AST/GOT	0.072	0.013	5.699	[0.045, 0.099]	<0.001 ***
Model 2—G2: FIB-4 (n = 90, R^2^ = 0.322, Adj.R^2^ = 0.282, F = 7.986, *p* < 0.001)					
Intercept	18.154	11.820	1.536	[−5.351, 41.660]	0.128
NLR	−0.134	0.082	−1.628	[−0.297, 0.030]	0.107
MLR	6.214	4.340	1.432	[−2.417, 14.845]	0.156
ALT/GPT	−0.405	0.081	−4.997	[−0.566, −0.244]	<0.001 ***
AST/GOT	0.225	0.040	5.623	[0.146, 0.305]	<0.001 ***
Age (years)	0.139	0.190	0.730	[−0.239, 0.516]	0.467
Model 3—G1: MDA (n = 20, R^2^ = 0.441, Adj.R^2^ = 0.292, F = 2.962, *p* = 0.055)					
Intercept	67.266	83.181	0.809	[−110.03, 244.56]	0.431
NLR	−38.588	29.564	−1.305	[−101.60, 24.43]	0.211
MLR	841.777	299.676	2.809	[203.03, 1480.52]	0.013 *
Glucose	0.183	0.294	0.622	[−0.444, 0.810]	0.543
AST/GOT	−1.166	0.845	−1.380	[−2.966, 0.634]	0.188

* OLS regression. β  =  unstandardized coefficient; SE  =  standard error. VIF verified for all models (VIF < 3.5). Residual normality confirmed by Shapiro–Wilk test. Significance: * *p* < 0.05; *** *p* < 0.001.

## Data Availability

Anonymized data are available from the corresponding authors upon reasonable request.

## Abbreviations

The following abbreviations are used in this manuscript:

8-iso-PGF2α	8-isoprostaglandin F2α
AASLD	American Association for the Study of Liver Diseases
AGEs	Advanced Glycation End-products
ALP	Alkaline Phosphatase
ALT	Alanine Aminotransferase (also GPT: Glutamate Pyruvate Transaminase)
AST	Aspartate Aminotransferase (also GOT: Glutamate Oxaloacetate Transaminase)
BD	Direct Bilirubin (Bilirubina Directă)
BMI	Body Mass Index
BT	Total Bilirubin (Bilirubina Totală)
CI	Confidence Interval
CV	Coefficient of Variation
EASD	European Association for the Study of Diabetes
EASL	European Association for the Study of the Liver
EASO	European Association for the Study of Obesity
ELISA	Enzyme-Linked Immunosorbent Assay
FIB-4	Fibrosis-4 Index
G1	Group 1 (MASLD with Type 2 Diabetes Mellitus)
G2	Group 2 (Control—MASLD without T2DM)
GGT	Gamma-Glutamyl Transferase
HbA1c	Glycated Hemoglobin (Hemoglobin A1c)
HCC	Hepatocellular Carcinoma
HDL	High-Density Lipoprotein Cholesterol
HRP	Horseradish Peroxidase
IQR	Interquartile Range
MAFLD	Metabolic-Associated Fatty Liver Disease (former nomenclature)
MASLD	Metabolic Dysfunction-Associated Steatotic Liver Disease
MASH	Metabolic Dysfunction-Associated Steatohepatitis
MCV	Mean Corpuscular Volume (also VEM: Volum Eritrocitar Mediu)
MDA	Malondialdehyde
MLR	Monocyte-to-Lymphocyte Ratio
MoMF	Monocyte-Derived Macrophage
NADPH	Nicotinamide Adenine Dinucleotide Phosphate
NAFLD	Non-Alcoholic Fatty Liver Disease (former nomenclature)
NASH	Non-Alcoholic Steatohepatitis (former nomenclature)
NLR	Neutrophil-to-Lymphocyte Ratio
OLS	Ordinary Least Squares
PKC	Protein Kinase C
PLR	Platelet-to-Lymphocyte Ratio
RDW	Red Cell Distribution Width
ROS	Reactive Oxygen Species
SD	Standard Deviation
SE	Standard Error
SMD	Standardized Mean Difference
SPSS	Statistical Package for the Social Sciences
T2DM	Type 2 Diabetes Mellitus
TBARS	Thiobarbituric Acid Reactive Substances
TMB	3,3′,5,5′-Tetramethylbenzidine substrate
VIF	Variance Inflation Factor
